# Editorial: Nervous regeneration and functional recovery in the central and peripheral nervous systems: diagnostic methods, gene/cell therapies, and interventions

**DOI:** 10.3389/fnmol.2025.1555390

**Published:** 2025-02-04

**Authors:** Dipa Natarajan, Ryo Hotta, Naho Fujiwara, Silvia Perin

**Affiliations:** ^1^Kcat Enzymatic Pvt Ltd, Bangalore, India; ^2^Mass General Research Institute, Massachusetts General Hospital, Harvard Medical School, Boston, MA, United States; ^3^School of Medicine, Juntendo University, Tokyo, Japan; ^4^UCL GOS Institute of Child Health, London, United Kingdom

**Keywords:** neuropathies, gene/cell therapies, interventions, SCI, Hirschsprung disease, biomarker, mechanoreceptor, signaling pathways

Significant progress has been made in comprehending neuropathies, with recent advancements in discovering remedies, refining analytical and diagnostic methods, and facilitating functional recoveries with gene and cell therapies and other complementary and alternative medicines.

The purpose of this Research Topic was to showcase new methods and gene/cell-based therapies that extend our diagnostic abilities and could potentially lead to functional recovery of the nervous system. This Research Topic includes reviews, original research and a brief research report where the topics cover newly developed methods for complex conditions such as Spinal Cord Injury (SCI) and Hirschsprung (HSCR) disease, include conventional methodology as well as molecular techniques to understand complementary and alternative medicines with promising progress.

For instance, Wang et al., used an iridoid rich fraction from *Valeriana jatamansi* Jones, a Chinese medicine for digestive disturbances and insomnia, to investigate its therapeutic role and mechanism of action *in vivo* using a rat model of SCI as well *in vitro* using PC12 cells. Their results indicate that IRVJ helps axonal regeneration post SCI and increases motor function recovery *in vivo* and improves cell survival and axonal regeneration *in vitro*. This occurs via the phosphoinositide-3-kinase (PI3K)/protein kinase B (Akt) signaling pathway, which is lost following the addition of its inhibitor, LY294002. Although more work is needed, IRVJ could develop into a potential drug for SCI patients.

Li et al., have given a detailed review of the role of Wnt signaling pathway in SCI, including its clinical application and future research directions. Wnt signaling plays a significant part in SCI recovery due to its neural stem cell proliferation and differentiation abilities, spinal inflammation modulation and anti-apoptotic actions. However, not much is known about the effects of long-term therapy and safety; in fact, since it is an important molecule affecting many processes and interacting with several signaling pathways, it cannot be taken single handedly as a treatment for SCI patients.

Therefore, to obtain functional recovery, an effective treatment must stem from the collaboration of many fields such as molecular medicine (with anti-inflammatory and immune modulatory methods), tissue engineering (with the use of different biomaterials) and perhaps precision medicine.

As a follow on from this study, a review by Ryu et al., has highlighted a novel yet still not fully explored area of research into the cellular signaling pathways and biological mechanisms activated in the mammalian nervous system by various mechanical and electromagnetic stimuli. Although used extensively for rehabilitation treatments, not much is known about how these stimuli work at the molecular level to influence therapies for neuro-regeneration. Mechanical stretching has the potential to stimulate adult neurogenesis in the brain involving several signaling pathways such as MAPK, PKC, YAP/TAK, Rho-ROCK and others that are being explored such as the transient receptor potential (TRP) ion channel family expressed widely in the brain, spinal cord etc, and purinergic receptors amongst others all of which respond to mechanical stimulation. Significantly, ion channels Piezo 1 and 2, which play a crucial role in mechanotransduction and work by opening cation channels upon mechanical stimulation, have been the focus of study in the past decade. However, depending on activation methods, there are many varied outcomes in the CNS which also depend on cell types. A lot more detailed research is needed to understand the different modes of actions for the different mechanotransduction methods before they can be considered as a therapy.

In a separate study by Moneme et al., Piezo 1 was studied using the enteric neural crest-derived neurons *in vitro*. The effects of stretched and unstretched enteric neurons in the presence or absence of Piezo 1 inhibitors was evaluated using transcriptomics and bulk RNA sequencing. Their results showed that the activation of Piezo 1 inhibited neurite length and neuronal migration. Moreover, they also showed that Piezo 1 agonism impairs neuronal recovery after injury, suggesting that this ion channel continues to function in mature postnatal gut. In light of the fact that the gut functions by contracting and relaxing/distending (or stretching) and that the enteric neurons play a key role in this, it is very interesting and exciting to speculate that Piezo 1 has a role in the development of the enteric nervous system and in disease of the ENS. Further research into this will help toward understanding their role in enteric neuropathies as well as developing new therapies for these diseases.

Just as it is important to understand the molecular basis of diseases to develop specific therapies, it is equally important to diagnose the disease and its severity. Hirschsprung disease (HSCR) affects gut motility due to the absence of ganglia which contain neurons and glia leading to severe constipation and megacolon from birth onwards. Currently, this condition is diagnosed by means of biopsies from different regions of the colon, a rather invasive method especially for newborns. Sreepada et al., report a novel non-invasive method for early diagnosis to detect this disease. They analyzed microRNA (miRNA) from the urine of 5 HSCR patients and controls and observed differential expression in specific miRNAs using microarrays, database target searches and RT-QPCR. Despite the limited availability of suitable candidates for such study, this work shows promise, not only as a non-invasive early detection tool for HSCR, but also as a cost effective, faster and first step method for diagnosing these patients especially in newborn babies and young children.

This special article has covered a wide variety of topics and although many of the results presented are at a preliminary stage, they are cutting edge in their respective fields and show promise in the understanding, diagnosing and treatment of some of the neuropathies described here and, perhaps, to other diseases in the near future.

